# The church bridge project focus group results: African American perspectives of weight management programs to improve nutrition and physical activity behaviors

**DOI:** 10.1186/s40795-021-00442-2

**Published:** 2021-07-19

**Authors:** Jennifer L. Lemacks, Laurie S. Abbott, Tammy Greer, Renee Gunn, Ashley Bryant, LaShaundrea Bradford, Penny A. Ralston

**Affiliations:** 1 School of Kinesiology and Nutrition, The University of Southern Mississippi, 118 College Drive #5142, Hattiesburg, MS 39406-0001 USA; 2 College of Nursing, The Florida State University, Tallahassee, MS USA; 3 School of Psychology, The University of Southern Mississippi, Hattiesburg, MS USA; 4 Center on Better Health and Life for Undeserved Populations, The Florida State University, Tallahassee, FL USA

**Keywords:** Obesity, Church-based, African American, Minority health, Health disparities

## Abstract

**Background:**

The prevalence of obesity is disproportionately high among African Americans in the Southern US. More information is needed about factors that influence participation in nutrition and physical activity programs to promote healthy weight.

**Objective:**

The purpose of this study is to explore the weight management perceptions of young to middle aged adult African Americans.

**Methods:**

The Church Bridge Project intervention participants were recruited for two focus groups. Qualitative data were recorded, transcribed and a thematic content analysis was conducted to identify major themes.

**Results:**

Barriers included technology learning curve/burden and competing priorities. Facilitators included support, limited cost, convenience, and health. Participants perceived the term “weight management” program as overwhelming and defeating.

**Conclusion:**

The Church Bridge Project model confirmed social support and disease prevention as key factors for weight management. Further work should substantiate social support as a key factor to guide minority health efforts.

## Background

Obesity and chronic disease behavior management can be especially challenging in rural areas in the Deep South. Nearly half of the rural population in the United States (U.S) resides in the South [1], and in states such as Mississippi where a large percentage (79%) of counties are classified as rural [1], the percentage of obese residents (37.3%) is among the highest in the region [2]. Further, the state of Mississippi has greater Black population density (37.8%) compared with other Deep South states including Alabama (26.8%), Louisiana (32.6%), and Georgia (32.2%) [3] as well as a stark difference between the proportion of Black (29%) and White (10%) populations living below poverty levels [4]. Mississippi has a history of being medically underserved with a majority of the state designated as a primary care provider health professional shortage area [5]. Therefore, it is difficult to reach rural and remote populations with few available resources to support health education and management.

Community- and church-based programs have been effective in improving health outcomes and reaching minority and underserved populations. However, greater program attendance is key for better health outcomes as shown in previous church-based intervention research [6, 7]. Additionally, various types of social support, including general, religious and church social and instrumental support, have been associated with improved diet and physical activity behaviors among minority and rural communities [8, 9]. Social support may be crucial for engaging minorities in healthy behaviors compared to Caucasian counterparts [10] and technology may facilitate the support of health program participants. The supportive policies for the provision of telehealth/−medicine, including coverage and reimbursement, point toward the potential for Mississippi to be a model state for the use of technology in health behavior management programs [11]. Thus, the Church Bridge Project was developed and tested over a two-phase intervention among African Americans living in southern Mississippi [12]. This research was intended to support the need for adoption and implementation of weight management interventions, focusing on diet and physical activity education and behavior change, among minority communities in the South. The purpose of this study was to examine program acceptability, barriers, and facilitators of participation in a church-based weight management intervention among young to middle-aged adult African Americans in southern Mississippi. Additionally, this research explored perception about weight management programs and the use of technology in weight management programs.

## Methods

Focus group research was selected as the methodology for this study to gain collective information and rich understanding from a population that was reached (and either participated in the program or not) to enroll in a church-based weight management program that was not captured by our quantitative outcomes [13]. Focus groups were conducted after conclusion of the Church Bridge Project weight management intervention that was implemented in two churches from February 2017 to September 2017 [14]. The weight management intervention program included 12, 1-h group-based sessions that entailed a 20-min education component, 20-min motivational interviewing component and 15- to 20-min snack and social component. Eligible focus group participants were those determined at intervention enrollment to be overweight/obese based on BMI and self-identified as being African American between the ages of 18 and 50 years of age. The participants were recruited from a referral and enrollment database of potential participants developed for the intervention study [14]. The two types of participants recruited for the focus groups were classified as intervention “completers” and “non-participants.” “Completers” were defined as individuals who successfully completed the intervention program. “Non-participants” were defined as individuals eligible for the intervention, approached to participate and enrolled in the intervention but did not participate in the intervention sessions. Two focus groups were conducted for “completers” and for “non-participants” with a goal to recruit six to eight participants each. This sample size goal is in alignment with widely accepted sample size recommendations [15]. Potential participants for each focus group were contacted via telephone, invited to participate and given a verbal overview of the focus group purpose and processes. All study protocol and materials were approved by The University of Southern Mississippi Institutional Review Board. This study follows the guidelines set forth in the Belmont Report which require that human research subjects, a) will not participate in research unless and until they have given voluntary and informed consent, b) confidential information received from participants will be fully protected within the limits of the law, both during and after research is conducted, d) subjects may withdraw their participation at any time without penalty or loss of benefits to which they would otherwise be entitled, e) burdens put on research participants must be reasonable relative to anticipated benefits to themselves and to society as a whole, f) the selection of research participants must be equitable and defensible in terms of both the goals of the research and general considerations of fairness.

Upon arrival, focus group participants were assigned a random number to maintain confidentiality. Research staff reviewed the study consent information and obtained consent to participate after all questions were answered. The focus groups were conducted by two trained research assistants with one serving as a moderator and the second as a note-taker. A script was provided that served as a guide for the focus groups, which included study overview and procedures related to how the focus group was to be conducted and recorded. After providing informed consent, participants were informed that a digital voice recorder would record the conversation until the end of the session. Each focus group lasted approximately 1 h. The “completer” focus group included open-ended questions related to barriers and facilitators to participation in a weight management intervention and suggestions for program improvement, whereas the “non-participant” focus group included questions related to weight management program perceptions as well as barriers and facilitators to participation and the use of technology in a weight management intervention. Focus group participants were given a $25 gift certificate to a superstore at the completion of the session.

Our qualitative research process was guided by the four tenets of trustworthiness: credibility, confirmability, transferability, and dependability [16]. After the focus groups were completed, recordings were transcribed by one research assistant and reviewed for accuracy by a second. Data analysis was conducted using a series of steps [17] based on Braun and Clarke’s six-step framework for thematic content analysis [18]. The initial transcription and review of the data by two staff was considered to be first step to “become familiar with the data.” All notes or initial thoughts regarding the data were noted and recorded by each research staff. The next step involved the generation of initial codes which followed a theoretical and open coding approach. This included developed and modified codes assigned to pieces of text from the data that were related to the research question and interesting. The third step was to examine the initial codes and search for common themes across the codes that identified something significant about the data. These initial steps described were conducted independently by two staff. In the next step, the two staff met to compare and review the themes each one developed, discuss common and divergent themes, and review whether data supported the proposed themes and if there was any overlap between themes. Staff developed a final, refined single list of themes which were subsequently reviewed by the larger team of staff and investigators. Research staff and investigators together reviewed and discussed the draft themes and developed the final list of themes to include in the final results.

As data were analyzed, we also considered saturation of the data and defined saturation based on theoretical saturation or the emergence of new themes in the data [19]. Theoretical saturation for this project was “the point where no new codes are emerging in the data [19].” In alignment with grounded theory development guidance [19, 20], we also considered saturation as the justification for termination of analysis. In consideration of data saturation, we also considered prior theoretical work [14].

## Results

A total of 12 individuals (*n* = 6 “completers” and *n* = 6 “nonparticipants”) participated in two focus groups. Themes were identified based on barriers and facilitators to and suggestions for improvement of the weight management program as well as diet values and weight management program perceptions. Table 1 provides integrated results of main themes identified.

**Table 1:** Overview of the Main Themes Identified Regarding a Weight Management Program

Topic	Main Coded Themes
Barriers	Technology learning curve & burden
	Competing priorities
Facilitators and Motivators	Accountability
	Program support
	Interventionist support
	Technology support
	Convenience
	Limited cost
	Health benefits
Suggestions for Improvement	Easier diet and activity tracking
	Care with age labels
Perceptions of “health and diet”	Longevity
	Improved health
Perceptions of “weight management”	Overwhelm
	Defeated
Perceptions of “nutrition and physical activity”	Positive
	Approachable

### Barriers identified by “completers”

Two themes for weight management program barriers were identified by “completers”: 1) technology learning curve, and 2) competing priorities. Participants found it difficult to locate specific food items or activities in the mobile app to log their nutrition and physical activity behaviors. However, the technological barrier was viewed as something that could be overcome with time with one person stating “… but I got into how to do it and stuff, and I learned how to do it so it wasn’t too bad …” It was agreed by all participants that the main barriers to program attendance were work, family, and church obligations and holidays. These competing priorities were noted as a barrier related to physical or mental exhaustion rather than lack of time. One participant reported, “Once you get off of work, and you are kind of mentally drained.”

### Facilitators identified by “completers”

Program support/engagement was the major theme identified by “completers” as a motivator for participation in a weight management program. “Completers” defined program support/engagement as accountability, social support from group setting, support and engagement from interventionists, and technology support. Participants’ expressed that the program provided accountability to support program compliance. When asked if they would prefer group or individualized sessions, all preferred the group session format because of the perceived social support it provided. “Completers” also expressed appreciation for the program support and engagement received from interventionists, who were predominantly undergraduate and graduate students. Last, there was a general agreement of satisfaction with having access to recorded sessions among “completers” and it was viewed as a benefit when unable to physically attend a session. Table 2 includes themes and sample coded text.

**Table 2:** Sample Coded Text for Barriers and Facilitators of and Improvement for Weight Management Program among Completers and Non-participants

Main Coded Themes	Sample Coded Text
Barriers	
Technology learning curve & burden	Completer: “… Like if I ate cheese toast, I’d like to get something I guess close to cheese toast, but it wouldn’t just you know, that was the thing like that.” Non-participant: “I don’t know how great it would be for me because it requires consistency. I might start off tracking it, keeping up with it, but once I get busy or I get off track for any reason, I’m done ...”
Competing priorities	Completer: “It’s a lot of activity going on with the church and stuff … “ Non-participant: “My life is, I just have so much, you know ...”
Facilitators and Motivators	
Accountability	Completer: “But the fact that you have to come back, and someone’s expecting you to do that. Even if you don’t lose the weight, they are expecting you to eat right.” Non-participant: “For me, it would be great to learn healthier ways of doing things and to gain the support …”
Group Support	Completer: “It’s good to be as a group. One person doesn’t know everything, but [in] a group session we can communicate.”
Interventionist Support	Completer: “That kind of motivated us because y’all coming down here and that’s a big help to see somebody caring.”
Technology support	Completer: “I did do the YouTube each time missed … “ Non-participant: “It can coach you through it.”
Convenience	Non-participant: “When it will be offered, how often it will be offered, what would be the requirements?”
Limited Cost	Non-participant: “I know you’re gonna have to pay for everything. You’re gonna have to put something in. You have to pay to get healthy, it’s expensive … but at the same time it’s even more expensive if you add up all the doctor’s bills and all the medicines you have to buy.””
Health Benefits	Non-participant: “Healthier, you know, healthier. You know just feels good if you can come off your high blood pressure medicines, it just feels real good …”
Improvement Suggestion(s)	
Easier diet and activity tracking	Completer: “The tracker app was, cause I mean it’s hard to track anyway, but it’s even harder when you have to pick and choose or flip and flop...”
Care with Age Labels	Completer: “So maybe the age limit needs to be a little more because you don’t get a lot of participation from younger people. Our group was older people -- we’re not old, it was more like 40-somethings, not 20-somethings.”

### Suggestions for improvement identified by “completers”

“Completers” were asked for suggestions to improve the weight management program. The main suggestion for improvement was related to enhancing the food and physical activity tracking functions of the application. One participant stated it was the only problem, “The only thing I had a problem was the app, that was just the app thing, that, you know, that was pretty much the only …” The age criteria for participation in the program also caused confusion and potential enrollment delays. “Completers” suggested the age criteria be clarified and thought the call for “younger adults” was not for them; one person stated, “When I read the criteria, I thought I aged out of it.” Table 2 includes themes and sample coded text.

### “Non-participants’” weight management program perceptions

“Non-participants” were asked to identify thoughts and emotions associated with three different phrases that could be used to recruit participants for a weight management program. Those terms included “health and diet,” “weight management program,” and “nutrition and physical activity program.” Common thoughts regarding the terms “health and diet” included longevity and improved health. Many “non-participants” indicated the term “weight management program” evoked feelings of being overwhelmed or defeated. One participant stated, “Almost defeated before I start.” “Non-participants” agreed the term “nutrition and physical activity” was less intimidating and more positive and approachable, compared to “weight management.” One participant responded in agreement with another, “See like she said, different way of putting it. When you think about it, it’s the same thing, just putting different words... But nutrition and physical activity sounds a whole lot better than weight management.” Table 3 includes themes and sample coded text.

**Table 3:** Sample Coded Text for Perceptions of Various Terms to Promote Weight Management Programs among Non-participants

Perceptions of “Health and Diet”	
Longevity and Improved Health	“The better you eat, the better you feel, and the longer you’ll be around for your kids. I have high blood pressure, but I’m on two different blood pressure medications and that was a wake-up call for me. I’m gonna get fit, I’m gonna eat right, or I’m gonna die. So, having nutrition and diet is everything.”
Perceptions of “Weight Management Program”	
Overwhelm and Defeat	“Well for me, when I hear weight management, I think about all those negatives. I can no longer have, I feel deprived. I go to the negative part, instead of thinking about the positive part – what it’s going to do for me, the health and the finding myself again and all those different things. So, I don’t think about the positive. It’s all negative.”
Perceptions of “Nutrition and Physical Activity Program”	
Positive and Approachable	“It’s a little better than weight management because to me that sounds like exercise and eating healthy, the two … So when I look at the portions and eating healthy and exercise, the two, I think of that term.”

### “Non-participant” barriers and facilitators to and use of Technology in Weight Management Programs

Similar to “completers,” “non-participants” expressed that competing priorities would be the major barrier to participation, to include family, work, church, and volunteer obligations.

When asked what would motivate them to participate in a nutrition and physical activity program, limited cost and convenience were identified. Additional motivators identified were health benefits to include pre-existing health conditions and overall health status. Pre-existing conditions were chronic diseases, including high blood pressure and diabetes. Overall health status included both mental and physical health. One participant explained: “Right now, what’s motivating me is my health. The second thing motivating me is loss of self, because I look in the mirror and I go, ‘Who is that person?’ I don’t recognize her because I’ve always been smaller my entire life. It’s just the last 5 or 10 years that I’ve slowly continued to gain weight.” “Non-participants” expressed that if they were able to participate in a program, they would hope to gain program support as accountability to achieve health goals. “Non-participants” also voiced technology could promote participation by providing support via being motivational, assisting with coaching, and providing reminders. Others expressed concerns with feeling burdened by needing to be consistent with using technology. Table 2 includes themes and sample coded text.

## Discussion

This study examined perceptions about a weight management program delivered in a church-based setting that were useful for identifying factors related to the weight management intervention design and implementation. The themes gleaned from the focus groups suggested that the participants of the intervention, the “completers”, had positive perceptions about the program. However, use of technology seemed to be both a barrier and facilitator of program success. Competing priorities were discussed as barriers to weight management program participation among both “completers” and “non-participants.” Another theme involved the negative connotation associated with the “weight management program” terminology. Suggestions included framing the wording as a nutrition and physical activity program. Social support and personal health were also identified as key factors for the success of a weight management program.

The results of the focus groups indicate that careful considerations are needed when incorporating technology into health intervention research. Mobile technology was viewed as a way to facilitate motivation, session attendance, and social support; however, a predominant theme among “completers” was that the food log function of the mobile application was difficult to use. A review of articles about dietary assessment using mobile phones found that six of the seven included studies reviewed had high participant satisfaction regarding use of mobile phones for dietary assessment [21]. The results of two additional studies also support the general acceptance of mobile food record methods among adults, including a community sample [22, 23]. Prior research found that higher user satisfaction was associated with more accurate dietary intake reporting [24]; however, it was not related to perceived participant burden to remember and log food intake [23]. Additionally, Krebs & Duncan [25] found that 44.5% of surveyed users discontinued using a health-related app because it took too long to enter data. Thus, while the use mobile applications for dietary assessment may be perceived as a satisfactory mode of data collection for participants, it may not reduce the perceived burden of data collection, which has data integrity implications.

While mobile technology may not reduce data collection burden, the supplementation of behavior interventions with technology may assist with maintaining program intensity/contact and providing individualized participant support without increasing participant burden. A review indicated that technology-based weight loss interventions induced positive weight-related outcomes, enhanced social support and self-monitoring opportunities, and improvement in program adherence [26]. Our previous research as well as conclusions from another study [27] examining health-related virtual communities support the hypothesis that technology, by increasing convenience and access, can enhance perceived support, thus improving outcomes. As we found, simple strategies such as providing participants live-streamed access to sessions when they cannot physically attend may increase program support and maintain participant commitment to the program. Since previous church-based intervention research studies have concluded that greater program attendance positively influences health outcomes [6, 7], an important theme was that competing priorities serve as major barriers of program attendance by young to middle aged African American adults. Further research is needed to ascertain from the population what strategies might assist them with circumventing program participation barriers.

Various types of social support (general, religious and church social and instrumental support) have been associated with improved diet and physical activity behaviors among minority and rural communities [8, 9] and is crucial for engaging the population in healthy behaviors [10]. Preliminary work also identified that family support was associated with weight loss intentions among an African American population. A similar theme found in this study was that the “church family” was a motivating factor for program participants. However, research examining potentially beneficial health effects of perceived social support from church and group-based interventions is minimal.

An additional theme, disease management and prevention objectives were identified as motivating factors of weight management program participation. It was interesting to note that although “non-participants” voiced a common theme of the importance of participating in weight management programs for health and longevity, they were not sufficiently motivated to actually participate in the program when offered. The relevant themes from the focus group data and previous literature support the importance of shifting the conversation about obesity from body ideals towards health in a culturally appropriate manner [12]. Challenges associated with this goal include perceptions among African Americans that obesity in itself is not an indicator of poor health [28], health is independent of obesity status [29], and “bigger is healthier” [30]. Other African American focus group participants have also viewed body mass index negatively [31] which corroborates our results of African Americans’ preference to frame “weight management interventions” as “nutrition and physical activity interventions” to enhance healthy lifestyle determinants and diminish the focus on weight. This suggestion is supported by currently published concepts that individuals should focus on being healthy and not achieving a certain body weight ideal [31].

### Limitations and strengths

In alignment with the four tenets of trustworthiness of qualitive inquiry [16], our results were credible, transferable, dependable, and ultimately, confirmable. Credibility addresses the goodness of fit between the participants’ ideas and the researchers’ interpretation of those ideas. We used a multi-step approach that included research triangulation, or multiple observers, at various steps to define and confirm themes that represent the data and reduce potential biases. Code and theme development was also documented at every step and examined by multiple researchers during the refinement and finalization of themes. Transferability refers to the generalizability of the knowledge gained from the research. This study underpins the importance of addressing weight management from a disease prevention and preventive health perspective. Additionally, church-based programs, compared to traditional medical settings, may be advantageously equipped with social support to facilitate diet, physical activity and other health behavior change goals. While our results are limited to an African American population in rural, Mississippi and a small sample size, these findings are not completely isolated in the literature and may have implications for other populations, especially across the Deep South. We also only conducted one focus group for each group type. Our research staff felt that they had saturated the pool of potential participants with recruitment efforts and did not feel further recruitment would yield enough participants for an additional focus group. Our sample does uniquely represent a predominantly younger to middle aged African American population, which will be a crucial target for health education and behavior programs to go beyond the management of disease toward the reduction of disease risk and ultimately, health disparities.

Last, our research is both dependable and confirmable. Dependability refers to the research process in that it is clear and reproducible. Our steps are documented and in alignment with best practice guidelines for qualitative inquiry. Confirmability requires demonstration of how conclusions were reached which is met when credibility, transferability and dependability are achieved. Our study outlines a clear qualitative process for how themes were derived with multiple checkpoints and results have clear implications for weight management in a specified population, which may have implications for and guide research in other populations.

## Conclusion

The findings from this study may help improve the development of lifestyle interventions intended for implementation among underserved, rural populations. The language used to describe weight management programs may deter participants and should consider a focus on the key factors identified by the population of interest (ie. improving nutrition and physical activity behaviors to achieve weight loss) versus the weight loss itself. Technology may serve to alleviate certain barriers and introduce others. Careful consideration of the population to be served and understanding technology related barriers is recommended to ensure that the pros outweigh the cons of use.

## Data Availability

The datasets used and/or analysed during the current study are available from the corresponding author on reasonable request.
